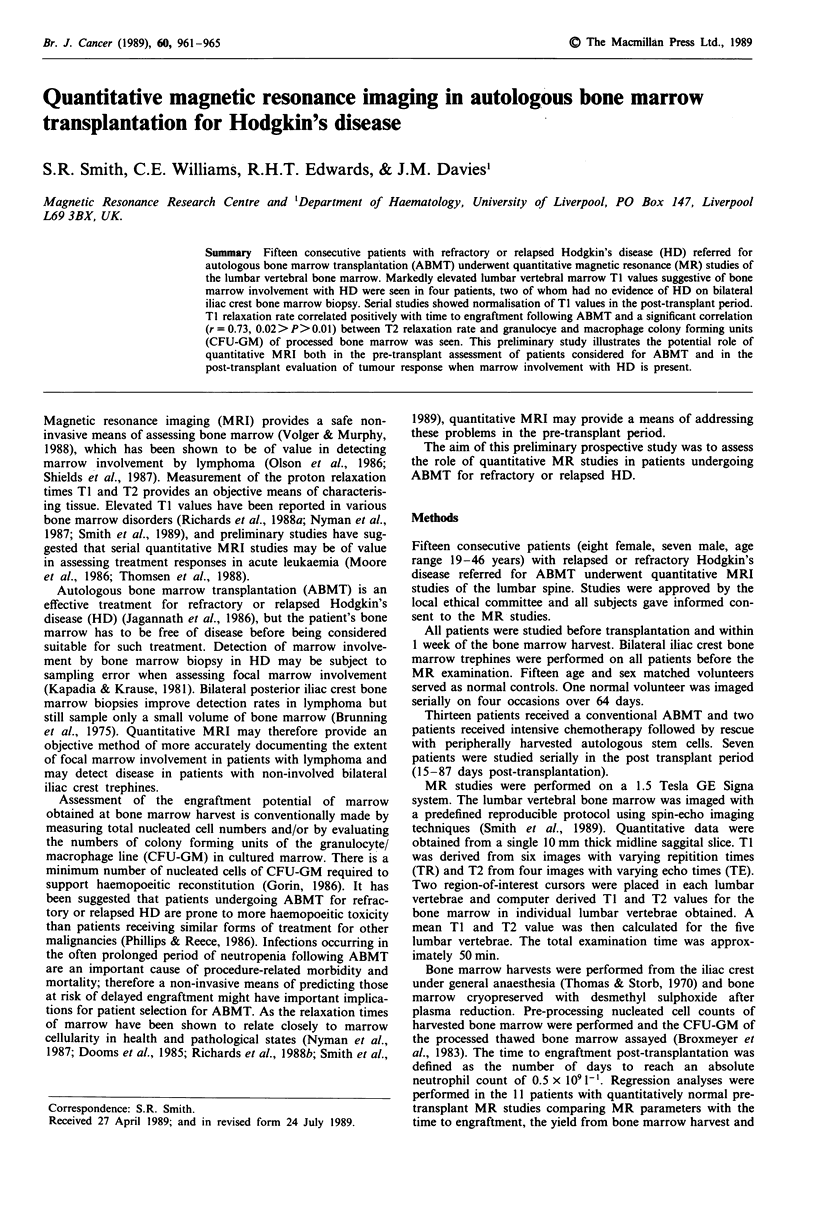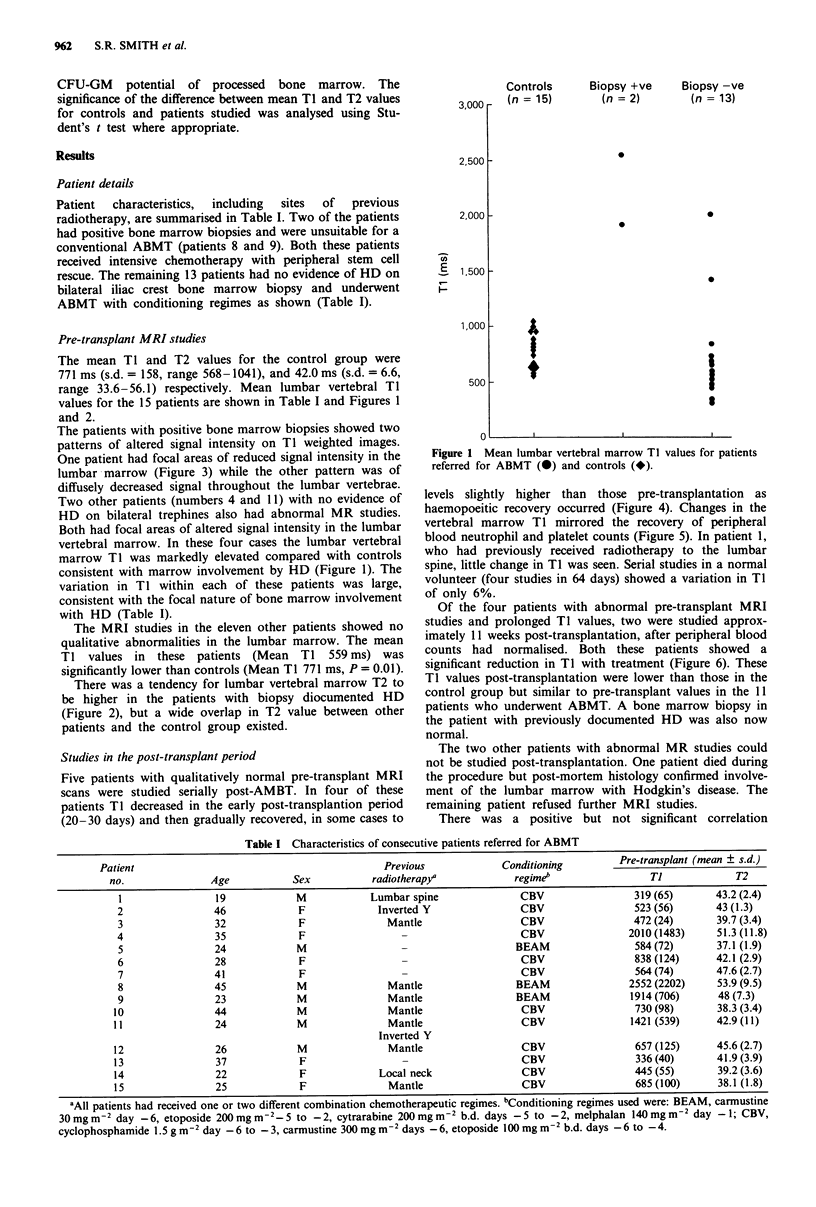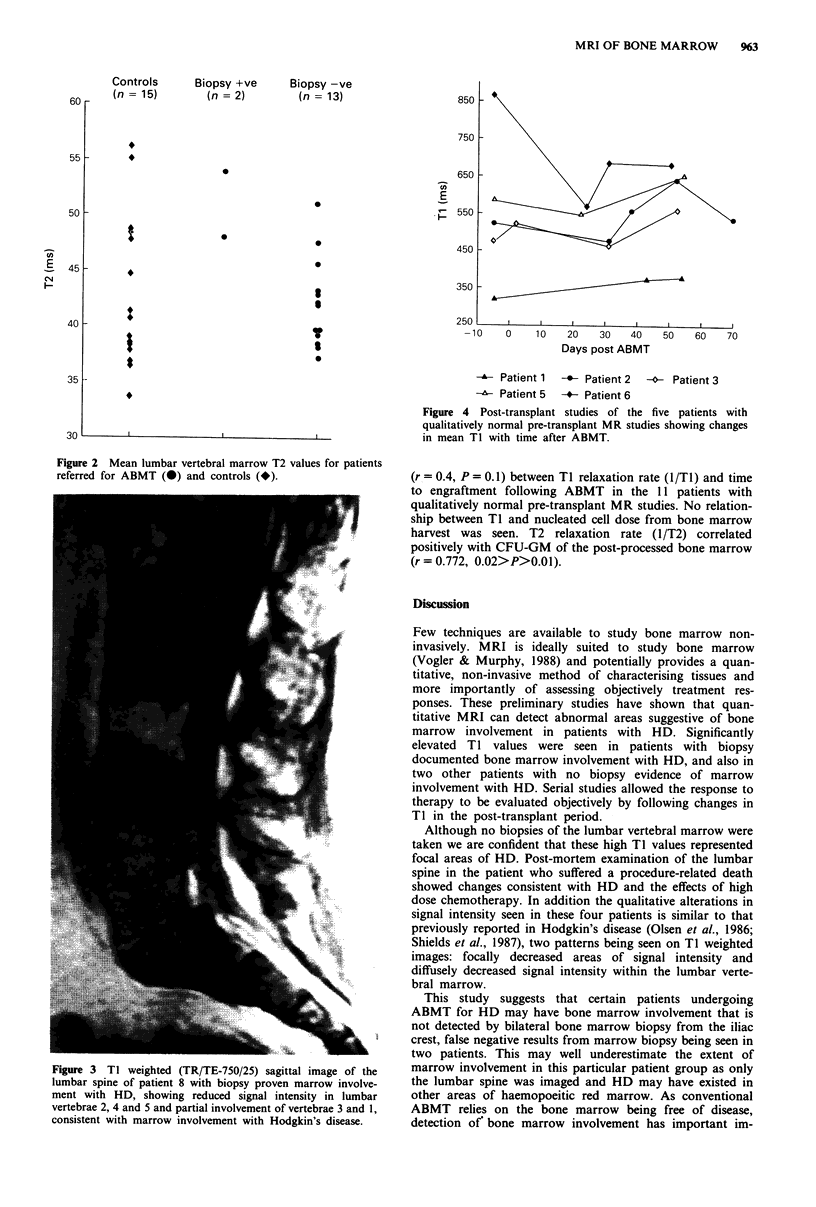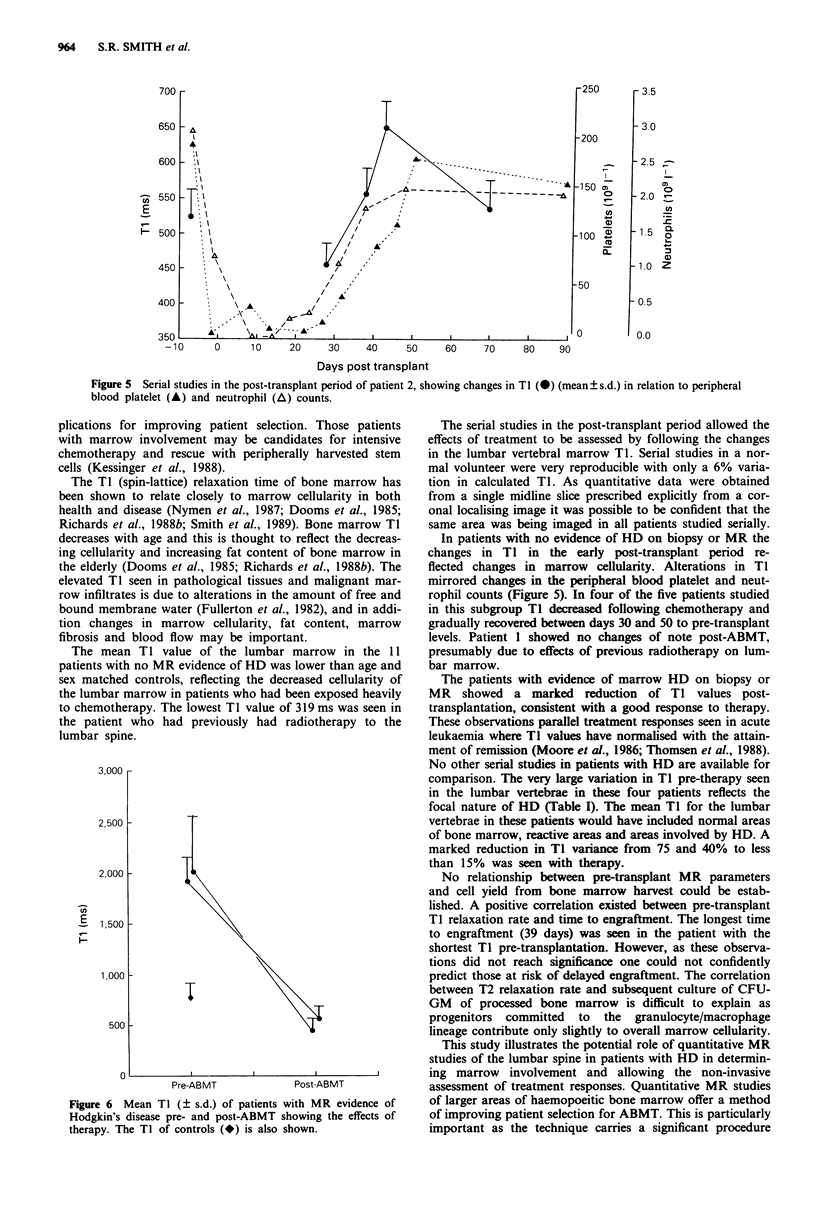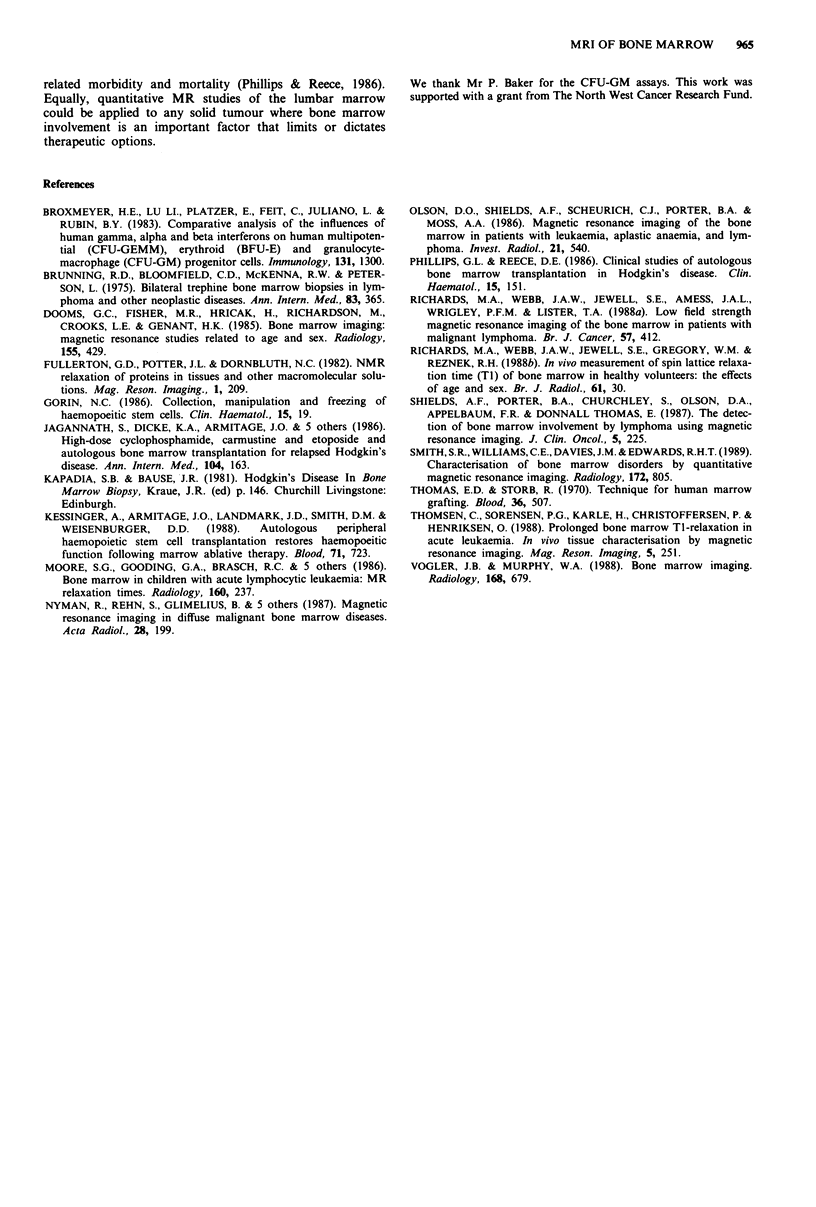# Quantitative magnetic resonance imaging in autologous bone marrow transplantation for Hodgkin's disease.

**DOI:** 10.1038/bjc.1989.398

**Published:** 1989-12

**Authors:** S. R. Smith, C. E. Williams, R. H. Edwards, J. M. Davies

**Affiliations:** Magnetic Resonance Research Centre, University of Liverpool, UK.

## Abstract

**Images:**


Br. J. Cancer (1989), 60, 961–965 © The Macmillan Press Ltd., 1989

### Quantitative magnetic resonance imaging in autologous bone marrow transplantation for Hodgkin's disease

S.R. Smith, C.E. Williams, R.H.T. Edwards, & J.M. Davies[1]

*Magnetic Resonance Research Centre and [1]Department of Haematology, University of Liverpool, PO Box 147, Liverpool L69 3BX, UK.*

**Summary** Fifteen consecutive patients with refractory or relapsed Hodgkin's disease (HD) referred for autologous bone marrow transplantation (ABMT) underwent quantitative magnetic resonance (MR) studies of the lumbar vertebral bone marrow. Markedly elevated lumbar vertebral marrow T1 values suggestive of bone marrow involvement with HD were seen in four patients, two of whom had no evidence of HD on bilateral iliac crest bone marrow biopsy. Serial studies showed normalisation of T1 values in the post-transplant period. T1 relaxation rate correlated positively with time to engraftment following ABMT and a significant correlation ($r = 0.73$, $0.02 > P > 0.01$) between T2 relaxation rate and granulocye and macrophage colony forming units (CFU-GM) of processed bone marrow was seen. This preliminary study illustrates the potential role of quantitative MRI both in the pre-transplant assessment of patients considered for ABMT and in the post-transplant evaluation of tumour response when marrow involvement with HD is present.

Magnetic resonance imaging (MRI) provides a safe non-invasive means of assessing bone marrow (Volger & Murphy, 1988), which has been shown to be of value in detecting marrow involvement by lymphoma (Olson *et al.*, 1986; Shields *et al.*, 1987). Measurement of the proton relaxation times T1 and T2 provides an objective means of characterising tissue. Elevated T1 values have been reported in various bone marrow disorders (Richards *et al.*, 1988*a*; Nyman *et al.*, 1987; Smith *et al.*, 1989), and preliminary studies have suggested that serial quantitative MRI studies may be of value in assessing treatment responses in acute leukaemia (Moore *et al.*, 1986; Thomsen *et al.*, 1988).

Autologous bone marrow transplantation (ABMT) is an effective treatment for refractory or relapsed Hodgkin's disease (HD) (Jagannath *et al.*, 1986), but the patient's bone marrow has to be free of disease before being considered suitable for such treatment. Detection of marrow involvement by bone marrow biopsy in HD may be subject to sampling error when assessing focal marrow involvement (Kapadia & Krause, 1981). Bilateral posterior iliac crest bone marrow biopsies improve detection rates in lymphoma but still sample only a small volume of bone marrow (Brunning *et al.*, 1975). Quantitative MRI may therefore provide an objective method of more accurately documenting the extent of focal marrow involvement in patients with lymphoma and may detect disease in patients with non-involved bilateral iliac crest trephines.

Assessment of the engraftment potential of marrow obtained at bone marrow harvest is conventionally made by measuring total nucleated cell numbers and/or by evaluating the numbers of colony forming units of the granulocyte/macrophage line (CFU-GM) in cultured marrow. There is a minimum number of nucleated cells of CFU-GM required to support haemopoeitic reconstitution (Gorin, 1986). It has been suggested that patients undergoing ABMT for refractory or relapsed HD are prone to more haemopoeitic toxicity than patients receiving similar forms of treatment for other malignancies (Phillips & Reece, 1986). Infections occurring in the often prolonged period of neutropenia following ABMT are an important cause of procedure-related morbidity and mortality; therefore a non-invasive means of predicting those at risk of delayed engraftment might have important implications for patient selection for ABMT. As the relaxation times of marrow have been shown to relate closely to marrow cellularity in health and pathological states (Nyman *et al.*, 1987; Dooms *et al.*, 1985; Richards *et al.*, 1988*b*; Smith *et al.*, 1989), quantitative MRI may provide a means of addressing these problems in the pre-transplant period.

The aim of this preliminary prospective study was to assess the role of quantitative MR studies in patients undergoing ABMT for refractory or relapsed HD.

### Methods

Fifteen consecutive patients (eight female, seven male, age range 19–46 years) with relapsed or refractory Hodgkin's disease referred for ABMT underwent quantitative MRI studies of the lumbar spine. Studies were approved by the local ethical committee and all subjects gave informed consent to the MR studies.

All patients were studied before transplantation and within 1 week of the bone marrow harvest. Bilateral iliac crest bone marrow trephines were performed on all patients before the MR examination. Fifteen age and sex matched volunteers served as normal controls. One normal volunteer was imaged serially on four occasions over 64 days.

Thirteen patients received a conventional ABMT and two patients received intensive chemotherapy followed by rescue with peripherally harvested autologous stem cells. Seven patients were studied serially in the post transplant period (15–87 days post-transplantation).

MR studies were performed on a 1.5 Tesla GE Signa system. The lumbar vertebral bone marrow was imaged with a predefined reproducible protocol using spin-echo imaging techniques (Smith *et al.*, 1989). Quantitative data were obtained from a single 10 mm thick midline saggital slice. T1 was derived from six images with varying repitition times (TR) and T2 from four images with varying echo times (TE). Two region-of-interest cursors were placed in each lumbar vertebrae and computer derived T1 and T2 values for the bone marrow in individual lumbar vertebrae obtained. A mean T1 and T2 value was then calculated for the five lumbar vertebrae. The total examination time was approximately 50 min.

Bone marrow harvests were performed from the iliac crest under general anaesthesia (Thomas & Storb, 1970) and bone marrow cryopreserved with desmethyl sulphoxide after plasma reduction. Pre-processing nucleated cell counts of harvested bone marrow were performed and the CFU-GM of the processed thawed bone marrow assayed (Broxmeyer *et al.*, 1983). The time to engraftment post-transplantation was defined as the number of days to reach an absolute neutrophil count of $0.5 \times 10^9 \, l^{-1}$. Regression analyses were performed in the 11 patients with quantitatively normal pre-transplant MR studies comparing MR parameters with the time to engraftment, the yield from bone marrow harvest and

Correspondence: S.R. Smith.
Received 27 April 1989; and in revised form 24 July 1989.

CFU-GM potential of processed bone marrow. The significance of the difference between mean T1 and T2 values for controls and patients studied was analysed using Student's *t* test where appropriate.

### Results

### *Patient details*

Patient characteristics, including sites of previous radiotherapy, are summarised in Table I. Two of the patients had positive bone marrow biopsies and were unsuitable for a conventional ABMT (patients 8 and 9). Both these patients received intensive chemotherapy with peripheral stem cell rescue. The remaining 13 patients had no evidence of HD on bilateral iliac crest bone marrow biopsy and underwent ABMT with conditioning regimes as shown (Table I).

### *Pre-transplant MRI studies*

The mean T1 and T2 values for the control group were 771 ms (s.d. = 158, range 568–1041), and 42.0 ms (s.d. = 6.6, range 33.6–56.1) respectively. Mean lumbar vertebral T1 values for the 15 patients are shown in Table I and Figures 1 and 2.

The patients with positive bone marrow biopsies showed two patterns of altered signal intensity on T1 weighted images. One patient had focal areas of reduced signal intensity in the lumbar marrow (Figure 3) while the other pattern was of diffusely decreased signal throughout the lumbar vertebrae. Two other patients (numbers 4 and 11) with no evidence of HD on bilateral trephines also had abnormal MR studies. Both had focal areas of altered signal intensity in the lumbar vertebral marrow. In these four cases the lumbar vertebral marrow T1 was markedly elevated compared with controls consistent with marrow involvement by HD (Figure 1). The variation in T1 within each of these patients was large, consistent with the focal nature of bone marrow involvement with HD (Table I).

The MRI studies in the eleven other patients showed no qualitative abnormalities in the lumbar marrow. The mean T1 values in these patients (Mean T1 559 ms) was significantly lower than controls (Mean T1 771 ms, $P = 0.01$).

There was a tendency for lumbar vertebral marrow T2 to be higher in the patients with biopsy diocumented HD (Figure 2), but a wide overlap in T2 value between other patients and the control group existed.

### *Studies in the post-transplant period*

Five patients with qualitatively normal pre-transplant MRI scans were studied serially post-AMBT. In four of these patients T1 decreased in the early post-transplantion period (20–30 days) and then gradually recovered, in some cases to

**Figure 1** Mean lumbar vertebral marrow T1 values for patients referred for ABMT (●) and controls (◆).

levels slightly higher than those pre-transplantation as haemopoeitic recovery occurred (Figure 4). Changes in the vertebral marrow T1 mirrored the recovery of peripheral blood neutrophil and platelet counts (Figure 5). In patient 1, who had previously received radiotherapy to the lumbar spine, little change in T1 was seen. Serial studies in a normal volunteer (four studies in 64 days) showed a variation in T1 of only 6%.

Of the four patients with abnormal pre-transplant MRI studies and prolonged T1 values, two were studied approximately 11 weeks post-transplantation, after peripheral blood counts had normalised. Both these patients showed a significant reduction in T1 with treatment (Figure 6). These T1 values post-transplantation were lower than those in the control group but similar to pre-transplant values in the 11 patients who underwent ABMT. A bone marrow biopsy in the patient with previously documented HD was also now normal.

The two other patients with abnormal MR studies could not be studied post-transplantation. One patient died during the procedure but post-mortem histology confirmed involvement of the lumbar marrow with Hodgkin's disease. The remaining patient refused further MRI studies.

There was a positive but not significant correlation

**Table I** Characteristics of consecutive patients referred for ABMT

| *Patient no.* | *Age* | *Sex* | *Previous radiotherapy*[a] | *Conditioning regime*[b] | *Pre-transplant (mean ± s.d.)* T1 | T2 |
|---|---|---|---|---|---|---|
| 1 | 19 | M | Lumbar spine | CBV | 319 (65) | 43.2 (2.4) |
| 2 | 46 | F | Inverted Y | CBV | 523 (56) | 43 (1.3) |
| 3 | 32 | F | Mantle | CBV | 472 (24) | 39.7 (3.4) |
| 4 | 35 | F | – | CBV | 2010 (1483) | 51.3 (11.8) |
| 5 | 24 | M | – | BEAM | 584 (72) | 37.1 (1.9) |
| 6 | 28 | F | – | CBV | 838 (124) | 42.1 (2.9) |
| 7 | 41 | F | – | CBV | 564 (74) | 47.6 (2.7) |
| 8 | 45 | M | Mantle | BEAM | 2552 (2202) | 53.9 (9.5) |
| 9 | 23 | M | Mantle | BEAM | 1914 (706) | 48 (7.3) |
| 10 | 44 | M | Mantle | CBV | 730 (98) | 38.3 (3.4) |
| 11 | 24 | M | Mantle<br>Inverted Y | CBV | 1421 (539) | 42.9 (11) |
| 12 | 26 | M | Mantle | CBV | 657 (125) | 45.6 (2.7) |
| 13 | 37 | F | – | CBV | 336 (40) | 41.9 (3.9) |
| 14 | 22 | F | Local neck | CBV | 445 (55) | 39.2 (3.6) |
| 15 | 25 | F | Mantle | CBV | 685 (100) | 38.1 (1.8) |

[a]All patients had received one or two different combination chemotherapeutic regimes. [b]Conditioning regimes used were: BEAM, carmustine 30 mg $m^{-2}$ day −6, etoposide 200 mg $m^{-2}$ −5 to −2, cytrarabine 200 mg $m^{-2}$ b.d. days −5 to −2, melphalan 140 mg $m^{-2}$ day −1; CBV, cyclophosphamide 1.5 g $m^{-2}$ day −6 to −3, carmustine 300 mg $m^{-2}$ days −6, etoposide 100 mg $m^{-2}$ b.d. days −6 to −4.

**Figure 2** Mean lumbar vertebral marrow T2 values for patients referred for ABMT (●) and controls (◆).

**Figure 3** T1 weighted (TR/TE-750/25) sagittal image of the lumbar spine of patient 8 with biopsy proven marrow involvement with HD, showing reduced signal intensity in lumbar vertebrae 2, 4 and 5 and partial involvement of vertebrae 3 and 1, consistent with marrow involvement with Hodgkin's disease.

**Figure 4** Post-transplant studies of the five patients with qualitatively normal pre-transplant MR studies showing changes in mean T1 with time after ABMT.

($r = 0.4$, $P = 0.1$) between T1 relaxation rate (1/T1) and time to engraftment following ABMT in the 11 patients with qualitatively normal pre-transplant MR studies. No relationship between T1 and nucleated cell dose from bone marrow harvest was seen. T2 relaxation rate (1/T2) correlated positively with CFU-GM of the post-processed bone marrow ($r = 0.772$, $0.02 > P > 0.01$).

### Discussion

Few techniques are available to study bone marrow non-invasively. MRI is ideally suited to study bone marrow (Vogler & Murphy, 1988) and potentially provides a quantitative, non-invasive method of characterising tissues and more importantly of assessing objectively treatment responses. These preliminary studies have shown that quantitative MRI can detect abnormal areas suggestive of bone marrow involvement in patients with HD. Significantly elevated T1 values were seen in patients with biopsy documented bone marrow involvement with HD, and also in two other patients with no biopsy evidence of marrow involvement with HD. Serial studies allowed the response to therapy to be evaluated objectively by following changes in T1 in the post-transplant period.

Although no biopsies of the lumbar vertebral marrow were taken we are confident that these high T1 values represented focal areas of HD. Post-mortem examination of the lumbar spine in the patient who suffered a procedure-related death showed changes consistent with HD and the effects of high dose chemotherapy. In addition the qualitative alterations in signal intensity seen in these four patients is similar to that previously reported in Hodgkin's disease (Olsen *et al.*, 1986; Shields *et al.*, 1987), two patterns being seen on T1 weighted images: focally decreased areas of signal intensity and diffusely decreased signal intensity within the lumbar vertebral marrow.

This study suggests that certain patients undergoing ABMT for HD may have bone marrow involvement that is not detected by bilateral bone marrow biopsy from the iliac crest, false negative results from marrow biopsy being seen in two patients. This may well underestimate the extent of marrow involvement in this particular patient group as only the lumbar spine was imaged and HD may have existed in other areas of haemopoeitic red marrow. As conventional ABMT relies on the bone marrow being free of disease, detection of bone marrow involvement has important im-

**Figure 5** Serial studies in the post-transplant period of patient 2, showing changes in T1 (●) (mean ± s.d.) in relation to peripheral blood platelet (▲) and neutrophil (Δ) counts.

plications for improving patient selection. Those patients with marrow involvement may be candidates for intensive chemotherapy and rescue with peripherally harvested stem cells (Kessinger *et al.*, 1988).

The T1 (spin-lattice) relaxation time of bone marrow has been shown to relate closely to marrow cellularity in both health and disease (Nymen *et al.*, 1987; Dooms *et al.*, 1985; Richards *et al.*, 1988*b*; Smith *et al.*, 1989). Bone marrow T1 decreases with age and this is thought to reflect the decreasing cellularity and increasing fat content of bone marrow in the elderly (Dooms *et al.*, 1985; Richards *et al.*, 1988*b*). The elevated T1 seen in pathological tissues and malignant marrow infiltrates is due to alterations in the amount of free and bound membrane water (Fullerton *et al.*, 1982), and in addition changes in marrow cellularity, fat content, marrow fibrosis and blood flow may be important.

The mean T1 value of the lumbar marrow in the 11 patients with no MR evidence of HD was lower than age and sex matched controls, reflecting the decreased cellularity of the lumbar marrow in patients who had been exposed heavily to chemotherapy. The lowest T1 value of 319 ms was seen in the patient who had previously had radiotherapy to the lumbar spine.

**Figure 6** Mean T1 (± s.d.) of patients with MR evidence of Hodgkin's disease pre- and post-ABMT showing the effects of therapy. The T1 of controls (◆) is also shown.

The serial studies in the post-transplant period allowed the effects of treatment to be assessed by following the changes in the lumbar vertebral marrow T1. Serial studies in a normal volunteer were very reproducible with only a 6% variation in calculated T1. As quantitative data were obtained from a single midline slice prescribed explicitly from a coronal localising image it was possible to be confident that the same area was being imaged in all patients studied serially.

In patients with no evidence of HD on biopsy or MR the changes in T1 in the early post-transplant period reflected changes in marrow cellularity. Alterations in T1 mirrored changes in the peripheral blood platelet and neutrophil counts (Figure 5). In four of the five patients studied in this subgroup T1 decreased following chemotherapy and gradually recovered between days 30 and 50 to pre-transplant levels. Patient 1 showed no changes of note post-ABMT, presumably due to effects of previous radiotherapy on lumbar marrow.

The patients with evidence of marrow HD on biopsy or MR showed a marked reduction of T1 values post-transplantation, consistent with a good response to therapy. These observations parallel treatment responses seen in acute leukaemia where T1 values have normalised with the attainment of remission (Moore *et al.*, 1986; Thomsen *et al.*, 1988). No other serial studies in patients with HD are available for comparison. The very large variation in T1 pre-therapy seen in the lumbar vertebrae in these four patients reflects the focal nature of HD (Table I). The mean T1 for the lumbar vertebrae in these patients would have included normal areas of bone marrow, reactive areas and areas involved by HD. A marked reduction in T1 variance from 75 and 40% to less than 15% was seen with therapy.

No relationship between pre-transplant MR parameters and cell yield from bone marrow harvest could be established. A positive correlation existed between pre-transplant T1 relaxation rate and time to engraftment. The longest time to engraftment (39 days) was seen in the patient with the shortest T1 pre-transplantation. However, as these observations did not reach significance one could not confidently predict those at risk of delayed engraftment. The correlation between T2 relaxation rate and subsequent culture of CFU-GM of processed bone marrow is difficult to explain as progenitors committed to the granulocyte/macrophage lineage contribute only slightly to overall marrow cellularity.

This study illustrates the potential role of quantitative MR studies of the lumbar spine in patients with HD in determining marrow involvement and allowing the non-invasive assessment of treatment responses. Quantitative MR studies of larger areas of haemopoeitic bone marrow offer a method of improving patient selection for ABMT. This is particularly important as the technique carries a significant procedure related morbidity and mortality (Phillips & Reece, 1986). Equally, quantitative MR studies of the lumbar marrow could be applied to any solid tumour where bone marrow involvement is an important factor that limits or dictates therapeutic options.

We thank Mr P. Baker for the CFU-GM assays. This work was supported with a grant from The North West Cancer Research Fund.

### References

BROXMEYER, H.E., LU LI., PLATZER, E., FEIT, C., JULIANO, L. & RUBIN, B.Y. (1983). Comparative analysis of the influences of human gamma, alpha and beta interferons on human multipotential (CFU-GEMM), erythroid (BFU-E) and granulocyte-macrophage (CFU-GM) progenitor cells. *Immunology*, **131**, 1300.

BRUNNING, R.D., BLOOMFIELD, C.D., McKENNA, R.W. & PETERSON, L. (1975). Bilateral trephine bone marrow biopsies in lymphoma and other neoplastic diseases. *Ann. Intern. Med.*, **83**, 365.

DOOMS, G.C., FISHER, M.R., HRICAK, H., RICHARDSON, M., CROOKS, L.E. & GENANT, H.K. (1985). Bone marrow imaging: magnetic resonance studies related to age and sex. *Radiology*, **155**, 429.

FULLERTON, G.D., POTTER, J.L. & DORNBLUTH, N.C. (1982). NMR relaxation of proteins in tissues and other macromolecular solutions. *Mag. Reson. Imaging.*, **1**, 209.

GORIN, N.C. (1986). Collection, manipulation and freezing of haemopoeitic stem cells. *Clin. Haematol.*, **15**, 19.

JAGANNATH, S., DICKE, K.A., ARMITAGE, J.O. & 5 others (1986). High-dose cyclophosphamide, carmustine and etoposide and autologous bone marrow transplantation for relapsed Hodgkin's disease. *Ann. Intern. Med.*, **104**, 163.

KAPADIA, S.B. & BAUSE, J.R. (1981). Hodgkin's Disease In *Bone Marrow Biopsy*, Kraue, J.R. (ed) p. 146. Churchill Livingstone: Edinburgh.

KESSINGER, A., ARMITAGE, J.O., LANDMARK, J.D., SMITH, D.M. & WEISENBURGER, D.D. (1988). Autologous peripheral haemopoietic stem cell transplantation restores haemopoeitic function following marrow ablative therapy. *Blood*, **71**, 723.

MOORE, S.G., GOODING, G.A., BRASCH, R.C. & 5 others (1986). Bone marrow in children with acute lymphocytic leukaemia: MR relaxation times. *Radiology*, **160**, 237.

NYMAN, R., REHN, S., GLIMELIUS, B. & 5 others (1987). Magnetic resonance imaging in diffuse malignant bone marrow diseases. *Acta Radiol.*, **28**, 199.

OLSON, D.O., SHIELDS, A.F., SCHEURICH, C.J., PORTER, B.A. & MOSS, A.A. (1986). Magnetic resonance imaging of the bone marrow in patients with leukaemia, aplastic anaemia, and lymphoma. *Invest. Radiol.*, **21**, 540.

PHILLIPS, G.L. & REECE, D.E. (1986). Clinical studies of autologous bone marrow transplantation in Hodgkin's disease. *Clin. Haematol.*, **15**, 151.

RICHARDS, M.A., WEBB, J.A.W., JEWELL, S.E., AMESS, J.A.L., WRIGLEY, P.F.M. & LISTER, T.A. (1988*a*). Low field strength magnetic resonance imaging of the bone marrow in patients with malignant lymphoma. *Br. J. Cancer*, **57**, 412.

RICHARDS, M.A., WEBB, J.A.W., JEWELL, S.E., GREGORY, W.M. & REZNEK, R.H. (1988*b*). *In vivo* measurement of spin lattice relaxation time (T1) of bone marrow in healthy volunteers: the effects of age and sex. *Br. J. Radiol.*, **61**, 30.

SHIELDS, A.F., PORTER, B.A., CHURCHLEY, S., OLSON, D.A., APPELBAUM, F.R. & DONNALL THOMAS, E. (1987). The detection of bone marrow involvement by lymphoma using magnetic resonance imaging. *J. Clin. Oncol.*, **5**, 225.

SMITH, S.R., WILLIAMS, C.E., DAVIES, J.M. & EDWARDS, R.H.T. (1989). Characterisation of bone marrow disorders by quantitative magnetic resonance imaging. *Radiology*, **172**, 805.

THOMAS, E.D. & STORB, R. (1970). Technique for human marrow grafting. *Blood*, **36**, 507.

THOMSEN, C., SORENSEN, P.G., KARLE, H., CHRISTOFFERSEN, P. & HENRIKSEN, O. (1988). Prolonged bone marrow T1-relaxation in acute leukaemia. *In vivo* tissue characterisation by magnetic resonance imaging. *Mag. Reson. Imaging*, **5**, 251.

VOGLER, J.B. & MURPHY, W.A. (1988). Bone marrow imaging. *Radiology*, **168**, 679.